# Supplementary material for: Colliding heavy nuclei take multiple identities on the path to fusion
Source: Nat Commun. 2023 Dec 2;14:7988. doi: 10.1038/s41467-023-43817-8 (PMC10693600; doi:10.1038/s41467-023-43817-8)
Supplement: Supplementary file 1 — Supplementary Information [file 41467_2023_43817_MOESM1_ESM.pdf]

Colliding heavy nuclei take multiple identities on the path to fusion

Supplementary Information

K.J. Cook[1,2*], D.C. Rafferty[1], D.J. Hinde[1], E.C. Simpson[1], M. Dasgupta[1], L. Corradi[3], M. Evers[1], E. Fioretto[3], D.Y. Jeung[1], N. Lobanov[1], D.H. Luong[1], T. Mijatović[4], G. Montagnoli[5], A.M. Stefanini[3] and S. Szilner[4]

[1]Department of Nuclear Physics and Accelerator Applications, Research School of Physics, The Australian National University, Canberra, 2601, ACT, Australia.
[2]Facility for Rare Isotope Beams, Michigan State University, East Lansing, 48824, Michigan, USA.
[3]Istituto Nazionale di Fisica Nucleare, Laboratori Nazionali di Legnaro, Legnaro, I-35020 Italy.
[4]Ruđer Bošković Institute, Zagreb, HR-10001, Croatia.
[5]Dipartimento di Fisica e Astronomia, Universita di Padova, via Marzolo 8, Padova, I-35131, Italy.

*Corresponding author. E-mail: kaitlin.cook@anu.edu.au;

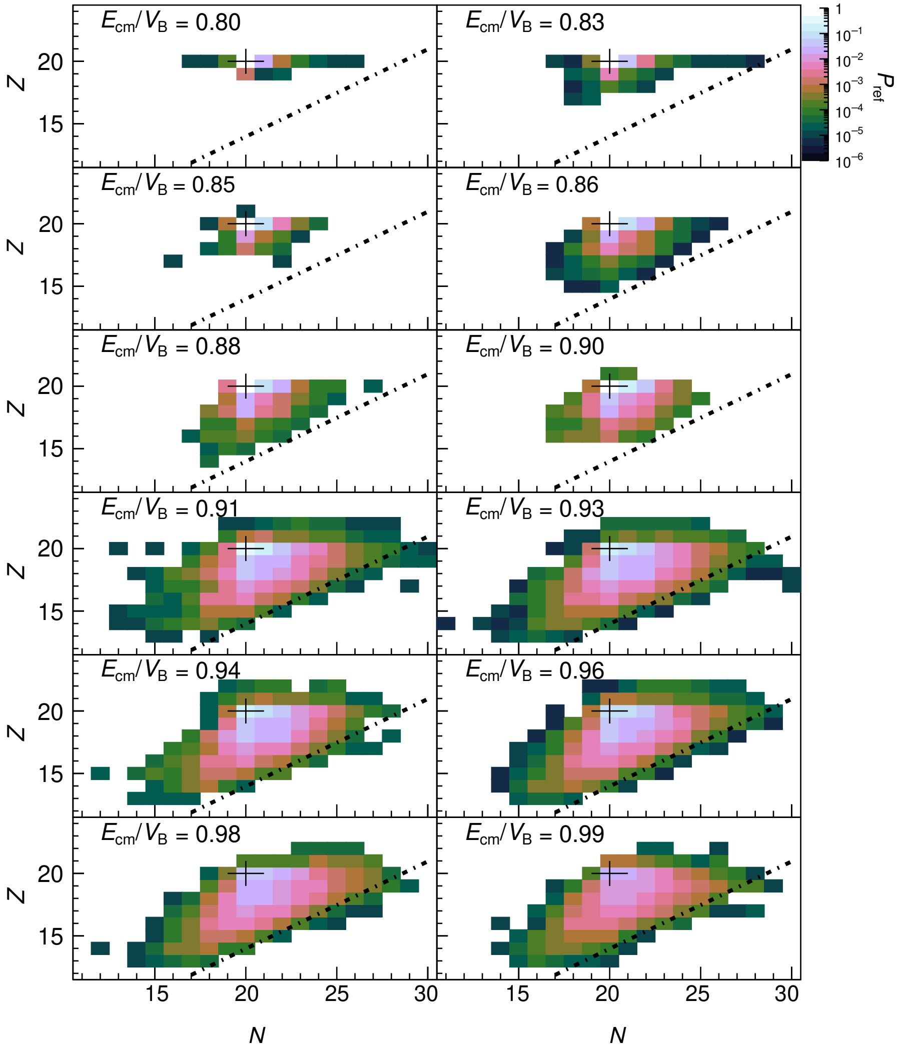

Supplementary Fig. 1 Z and N distribution of reflected nuclei produced in reactions of ^{40}Ca+^{208}Pb at $\theta_{lab} = 115°$ at measured energies from $E_{cm}/V_B = 0.81$ to 0.99. The common colour scale indicates the probability of forming each nucleus. The location of ^{40}Ca is indicated with the cross. Diagonal lines indicate the isospin asymmetry equal to that of the compound nucleus ^{248}No (N/Z = 1.43)

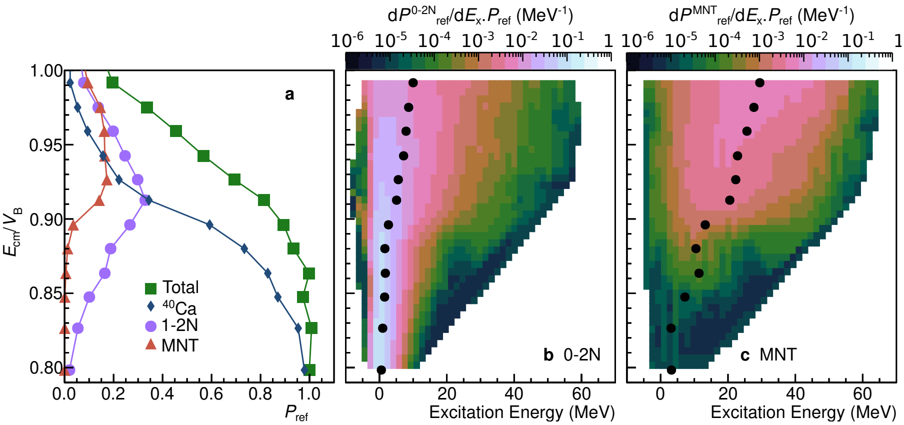

Supplementary Fig. 2 (a) Absolute probability of reflected flux for all reflected flux (green squares), (in)elastic scattering (^{40}Ca, blue diamonds), 1-2N transfer (purple circles), and multi-nucleon transfer (MNT, orange triangles) as a function of E/V_B. Statistical errors are smaller than the points. (b) Evolution of excitation energy with increasing E/V_B (decreasing separation) for the sum of in(elastic) scattering and 1-2N transfer, denoted "0-2N" and (c) for MNT. The points show the mean excitation energy at each measured energy. The colour axis indicates $P^{0-2\text{N}}_{\text{reflected}}/P_{\text{reflected}}$ and $P^{\text{MNT}}_{\text{reflected}}/P_{\text{reflected}}$, for panels (b) and (c) respectively. The data has been interpolated between measurement energies using Delaunay triangulation [1].

Supplementary Table 1 Centre-of-mass energies, barrier height V_B (incorporating the centrifugal potential for the ℓ value at 115°, distance of closest approach relative to the barrier and energy with respect to the barrier.

Energy (MeV)	V_B (θ_{lab} = 115°) (MeV)	$R_{min} - R_B$	E_{cm}/V_B
156.7	196.3	3.87	0.80
161.8	195.8	3.35	0.83
165.6	195.4	3.00	0.85
168.5	195.1	2.73	0.86
171.5	194.9	2.45	0.88
174.4	194.6	2.20	0.90
177.4	194.4	1.93	0.91
179.9	194.0	1.71	0.93
182.8	193.7	1.45	0.94
185.8	193.5	1.16	0.96
188.7	193.3	0.86	0.98
191.7	193.1	0.46	0.99

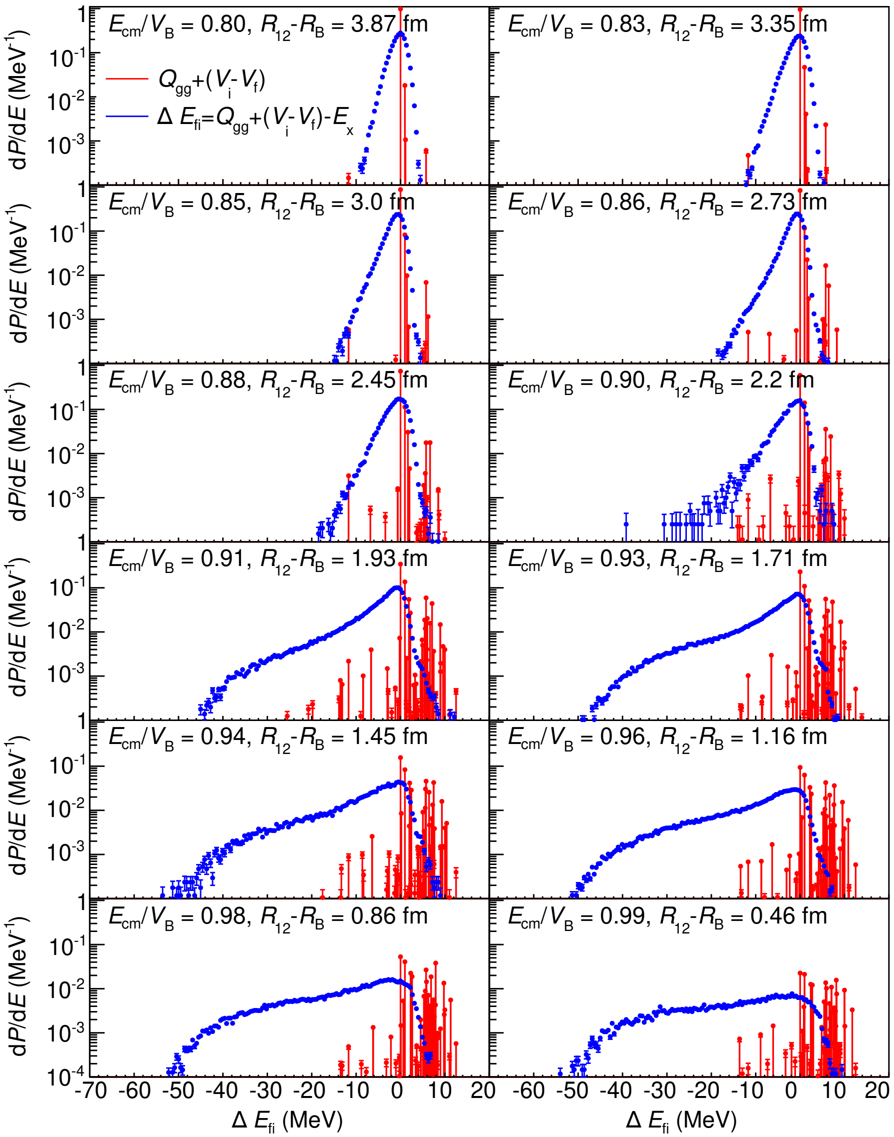

Supplementary Fig. 3 Distribution of kinetic energies with respect to the new potential following transfer at all measured energies. The blue curve shows the distribution including the measured excitation energy (i.e. $Q_{gg} + (V_i - V_f) - E_x$), where the red lines show the positions of ground-state to ground-state transfers (i.e. $Q_{gg} + (V_i - V_f)$). Error bars are statistical.

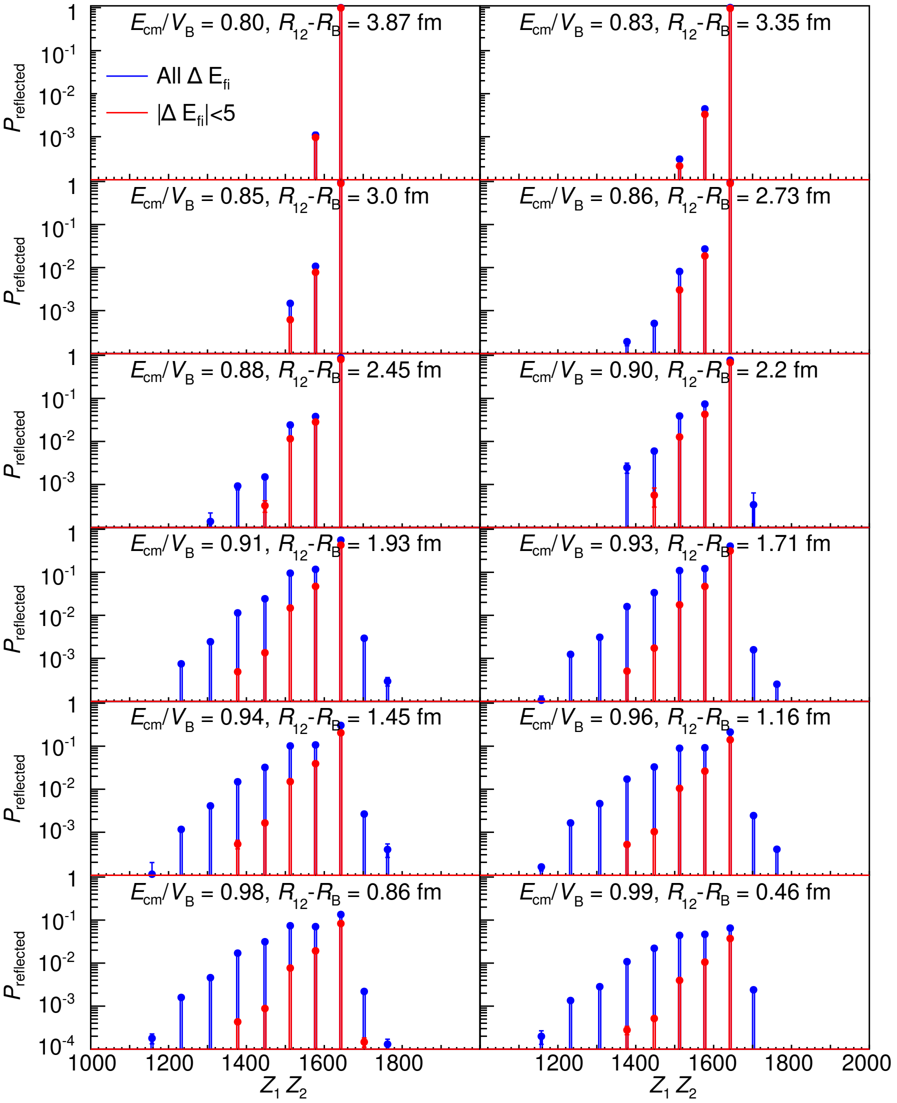

Supplementary Fig. 4 Distribution of reflected flux probabilities in exit channel Z_1Z_2 at all measured energies. The red curve shows the distribution including only those nuclides with energies with respect to the barrier similar to the entrance channel, i.e. $|\Delta E_{fi} < 5|$, where the blue lines show the distribution of Z_1Z_2 of the entire reflected flux. Error bars are statistical and mostly smaller than the size of each point.

Supplementary References

[1] Delaunay, B. Sur la sphère vide. a la mémoire de georges voronoï. *Bulletin de l'Académie des Sciences de l'URSS. Classe des sciences mathématiques et na* 793–800 (1934) .
